# Clinical Manifestations, Monitoring, and Prognosis: A Review of Cardiotoxicity After Antitumor Strategy

**DOI:** 10.3389/fcvm.2022.912329

**Published:** 2022-06-10

**Authors:** Wei Huang, Rong Xu, Bin Zhou, Chao Lin, Yingkun Guo, Huayan Xu, Xia Guo

**Affiliations:** ^1^Key Laboratory of Birth Defects and Related Diseases of Women and Children of Ministry of Education, Department of Radiology, West China Second University Hospital, Sichuan University, Chengdu, China; ^2^Laboratory of Molecular Translational Medicine, Key Laboratory of Birth Defects and Related Diseases of Women and Children (Sichuan University), Center for Translational Medicine, Ministry of Education, Clinical Research Center for Birth Defects of Sichuan Province, West China Second University Hospital, Sichuan University, Chengdu, China; ^3^Department of Hematology, West China Second University Hospital, Sichuan University, Chengdu, China

**Keywords:** cardiotoxicity, chemotherapy, immune checkpoint inhibitors (ICI), treatment measures, monitoring methods, cardiac magnetic resonance (CMR)

## Abstract

The development of various antitumor drugs has significantly improved the survival of patients with cancer. Many first-line chemotherapy drugs are cytotoxic and the cardiotoxicity is one of the most significant effects that could leads to poor prognosis and decreased survival rate. Cancer treatment include traditional anthracycline drugs, as well as some new targeted drugs such as trastuzumab and ICIs. These drugs may directly or indirectly cause cardiovascular injury through different mechanisms, and lead to increasing the risk of cardiovascular disease or accelerating the development of cardiovascular disease. Cardiotoxicity is clinically manifested by arrhythmia, decreased cardiac function, or even sudden death. The cardiotoxicity caused by traditional chemotherapy drugs such as anthracyclines are significantly known. The cardiotoxicity of some new antitumor drugs such like immune checkpoint inhibitors (ICIs) is also relatively clear and requiring further observation and verification. This review is focused on major three drugs with relatively high incidence of cardiotoxicity and poor prognosis and intended to provide an update on the clinical complications and outcomes of these drugs, and we innovatively summarize the monitoring status of survivors using these drugs and discuss the biomarkers and non-invasive imaging features to identify early cardiotoxicity. Finally, we summarize the prevention that decreasing antitumor drugs-induced cardiotoxicity.

## Introduction

Antitumor drugs are essential for cancer treatment. These drugs, especially various chemotherapy and immune checkpoint inhibitors (ICIs), have been rapidly developed and used extensively, resulting in the significant increase in the survival rate of patients with cancer. However, how to improve the 5-year survival rate and quality of long-term life of these patients after chemotherapy is a huge challenge that clinicians need to face in the long-term treatment/remission cycle.

Cardiotoxicity is served as one of the main factors that affected the quality of life and prognosis of cancer patients (1). Different types of chemotherapeutic drugs and ICIs currently used in different clinical conditions, cumulative doses, and treatment options may cause different effects of myocardial damage (2). Clinically, cardiotoxicity may be asymptomatic for a long time or manifest various symptoms such as arrhythmia, decreased systolic function, and/or myocarditis. These symptoms may occur immediately after drug administration or occur in months or years later (3). In some patients, cardiotoxicity could even affect the choice of cancer treatment strategy. Once the decreased cardiac function or other significant signs occur, patients can only receive poorly effective alternative drugs, or the treatment strategy may be terminated (2, 4, 5). Hence, cardiotoxicity is apparently severe, causing more uncertainty to the treatment of cancer.

Cardiotoxic complications are the main cause of mortality, and childhood cancer survivors have higher risk of cardiotoxicity (6). Compared with the general population, childhood cancer survivors have a 15-fold increase in the risk of congestive heart failure (CHF) and a 7-fold increase in the risk of premature death (7). Therefore, continuous monitoring of heart function throughout the entire cancer treatment course contributes to detecting myocardial damage, and timely intervention measures can prevent or even reverse cardiac dysfunction progression.

In this manuscript, we will review the clinical complications and outcomes of several traditional chemotherapy agents and newer targeted cancer therapies that may cause high incidence of cardiotoxicity and poor prognosis. We innovatively summarize the monitoring status of survivors using these drugs and discuss the biomarkers and non-invasive imaging features to identify early cardiotoxicity. Finally, we will summarize the prevention that decreasing antitumor drugs-induced cardiotoxicity.

## Clinical Manifestations and Prognosis

### Anthracyclines

Anthracyclines are one of the most widely used antitumor drugs. Approximately 1 million patients with cancer undergo anthracycline treatment each year. Generally, it is used clinically in many blood system and solid malignant tumors (3, 8). Anthracyclines include doxorubicin, which is isolated from the bacteria of the genus Streptomyces. Hence, they possess antibiotic properties and have become one of the most effective chemotherapy treatments ever (8).

Myocardial damage caused by anthracyclines have been confirmed by many studies and are generally recognized and accepted in clinical practice. From the initial oncology diagnosis, more than half of patients who received anthracycline therapy manifested with cardiac abnormalities after 10–20 years later (9). Of these patients, approximately 5% patients developed with CHF. Within 20 years after diagnosis, roughly 40% of patients experienced with arrhythmia (2). The American Society of Clinical Oncology has reported the high-risk indicators of cardiac dysfunction in adult cancer survivors, such indicators include the high-dose use of anthracyclines (doxorubicin ≥ 250 mg/m^2^, epirubicin ≥ 600 mg/m^2^) or the use of low-dose anthracyclines (doxorubicin ≤ 250 mg/m^2^ and epirubicin ≤ 600 mg/m^2^) accompanied with smoking, hypertension, diabetes, obesity, age over 60 years old, and/or other risk factors (1).

For survivors with previous anthracycline therapy, the asymptomatic stage is usually characterized by left ventricular (LV) wall thinning, LV diameter increase, and subsequent LV wall stress increase, which is similar to dilated cardiomyopathy (7). Anthracycline cardiotoxicity is related to the cumulative dose (4, 7, 9, 10). Meanwhile, the dose threshold of anthracyclines causing heart failure (HF) has been confirmed to become lower in nearly 30 years of research. According to the initial research, the dose threshold of doxorubicin-induced HF is 400 mg/m^2^ (11, 12). Without other influencing factors, the incidence of HF increases to 5, 26, and 48% when the cumulative anthracycline doses are 400, 550, and 700 mg/m^2^, respectively. However, a subsequent study suggested that the dose threshold for HF is lower (13). In a prospective analysis of clinical trials for patients with breast cancer and lung cancer, 9% of the study participants with a cumulative doxorubicin dose of 250 mg/m^2^ developed cardiac dysfunction. When doxorubicin was administered at a cumulative dose of 350 or 450 mg/m^2^, the incidence of cardiac dysfunction increased to 18 and 38%, respectively. According to a recent cardiomyopathy screening guideline, the high-risk cumulative anthracycline dose threshold for childhood cancer survivors is 250 mg/m^2^ (5, 7). But in other reports, the threshold of long-term cardiomyopathy risk with mitoxantrone is considerably lower than 250 mg/m^2^ in childhood (14). Therefore, the safe dose of anthracyclines remains unestablished.

Notably, some cases exhibited sudden HF during the course of cancer treatment. In a case provided by Saro, a 14-year-old patient with AML underwent three intensive treatments containing mitoxantrone (12 mg/m^2^/dose per day; four doses in total) after two cycles of remission induction. On day 3 of the second intensive treatment phase (the phase containing mitoxantrone), the patient developed febrile tachycardia and high oxygen demand. Echocardiogram showed that the LV was enlarged. Compared with the cardiac function before chemotherapy (ejection fraction, 60%; fractional shortening, 32%), the LV function was moderately to severely decreased (EF, 28%; SF, 14%) (4). Although the incidence of mitoxantrone-induced cardiotoxicity is low, symptoms may vary rapidly over a short period.

### Immune Checkpoint Inhibitors

The use of immune checkpoint inhibitor (ICI) was an important advance in the field of cancer therapy in the past decade. The anti-CTLA-4 antibody ipilimumab was the first ICI approved by the US Food and Drug Administration (FDA). Subsequently, pembrolizumab, durvalumab, and cemiplimab-rwlc have been approved for clinical treatment (15). ICI exhibits an antitumor effect by inhibiting the key regulators of immunotolerance hijacked by tumor cells. ICI is mostly used for treating various cancers, including melanoma (unresectable or metastatic disease and adjuvant therapy), metastatic non-small cell and small cell lung cancer, and locally advanced or metastatic squamous cell carcinoma of the skin. In patients with stage III (unresectable) or stage IV melanoma, treatment with ipilimumab can increase the median survival from 6.4 to 10.1 months (16). However, ICI use may cause immune-related adverse events (IRAEs) affecting multiple organs, such as the colon, lung, liver, skin, pituitary, thyroid, and heart. Multiple-organ dysfunction commonly occurs in therapies combined with ICIs (17). Wolchok et al. reported that ICIs combination therapy is significantly associated with adverse events (96%), and grade 3 or 4 adverse events occurred in 59%, treatment-related adverse events that led to the discontinuation of therapy occurred more frequently with combination therapy than with either monotherapy (the severity of adverse events was graded according to the National Cancer Institute Common Terminology Criteria for Adverse Events, version 4.0.) (18).

For cardiotoxicity, some research reported the incidence of cardiac IRAEs is low (<1%), but the prognosis extremely poor especially life-threatening fulminant myocarditis, and the median time from the first exposure of ICI to the onset of myocarditis is 30 days (19–24). In addition, left ventricular systolic dysfunction (LVSD) has an incidence rate of approximately 49–79%, which does not necessarily exist at the same time as ICI myocarditis. More notably, a previous study reported 16 serious adverse cardiovascular events related to ICI but 6 (38%) of them had a normal LVEF, which indicated patients with a normal ejection fraction may still develop ICI-related myocarditis (21). Furthermore, ICI-related myocarditis is associated with other IRAEs. In previous studies, among patients with ICI myocarditis, 25% had concomitant myositis and 10–11% had concomitant myasthenia gravis, which indicate that patients receiving ICI who present with myositis or myasthenia gravis should be assessed for ICI-associated myocarditis (20, 22, 25). A recent retrospective study proposed demographic risk factors for ICI cardiotoxicity. According to review 538 medical records of patients who underwent immunotherapy, Brumberger et al. found that there was a significantly higher percentage of women experiencing cardiac events compared to men (8.1 vs. 2.9%; P = 0.011) as well as a higher percentage of African Americans with cardiac events than Caucasians with cardiac events (12 vs. 4%; P = 0.02) (26). Patients undergoing treatment with Pembrolizumab (n = 243) had higher cardiac events rates compared to Nivolumab (n = 220) (7 vs. 4%) (26). The risk of myocarditis and the mortality rate are higher when ICI is combined with other drugs that also have cardiotoxic effects or more than two ICIs treatment (20, 22, 27). Salem et al. retrospectively analyzed 32 patients with myocarditis who received ICI combination therapy (anti-CTLA-4 plus anti-PD-1 or anti-PD-L1 therapy), and 21 (66%) of them had died (20). Therefore, once ICI-related myocarditis is suspected during treatment, patients need to terminate ICI treatment immediately and permanently because of the high mortality risk for the second treatment (28). In addition, ICI-related cardiotoxicity events also include pericardial disease, which can occur alone or association with myocarditis. The pericardial disease mostly affects patients with lung cancer. The median time from the first exposure of ICI to the onset of pericardial disease is approximately 30 days, and the mortality rate is roughly 21% (20). The ICI combination therapy also reportedly leads to some complex complications, such as vasculitis, various arrhythmia types, and acute coronary syndrome (ACS) (27); however, this matter has not yet been investigated epidemiologically.

### Trastuzumab

Currently, breast cancer is the most common malignancy among women worldwide, with over 2 million new cases diagnosed in 2018 (29). Human epidermal growth factor receptor 2 (HER2) protein overexpression occurs in approximately 20–25% of breast cancer cases and is related to aggressive tumor behavior (3, 9, 30). Trastuzumab is a recombinant humanized IgG1 monoclonal antibody that can selectively bind to HER2. It is often used in breast cancer chemotherapy for HER2 overexpression treatment and combined with anthracyclines. Trastuzumab combined with another chemotherapeutic drug yields a significant effect on HER2-positive breast cancer, but it can also inhibit tumor growth when used alone.

A study focusing on HER2 overexpression proved that trastuzumab combination therapy is effective and that its relative risk of death and the recurrence rate were reduced by 20 and 51%, respectively (31). However, cardiotoxicity is the main adverse event after trastuzumab treatment (30). Patients may present with myocardial injury, resulting in a poor prognosis including the risk of sudden death, especially when combined with anthracyclines. The most common clinical manifestation is the asymptomatic drop of left ventricular ejection fraction (LVEF) (3, 31, 32). Dennis et al. showed that the prevalence of New York Heart Association classification (NYHA) grade III or IV cardiac dysfunction is about 16% in patients receiving combination drug therapy comprising anthracyclines, cyclophosphamide, and trastuzumab, of which 8% of them developed cardiac dysfunction and stopped trastuzumab (31). Besides, trastuzumab-related cardiotoxicity is related to a long-term significant damage of the cardiopulmonary function. Cardiopulmonary function damage causes an increased risk of delayed cardiovascular disease in HER2-positive breast cancer survivors. In an 8-year follow-up study, the LVEF of approximately 40% of patients with breast cancer was reduced by 10% after 7 years of trastuzumab discontinuation that indicated these patients with cardiotoxicity. Otherwise, compared with those without cardiotoxicity evidence, these patients had a significantly reduced longitudinal strain and peak oxygen uptake (32).

Cardiotoxicity also been reported when trastuzumab is used in combination with other drugs even without anthracycline. A recent study compared two types of neoadjuvant chemotherapy for HER2 breast cancer and showed that the incidence of cardiac events was 7.7% among patients who received trastuzumab plus docetaxel and carboplatin (TCH) after 9 years’ follow-up. During chemotherapy or up to 1 year after chemotherapy, 4.6% patients in the TCH group developed early-stage CHF (33).

**FIGURE 1:**
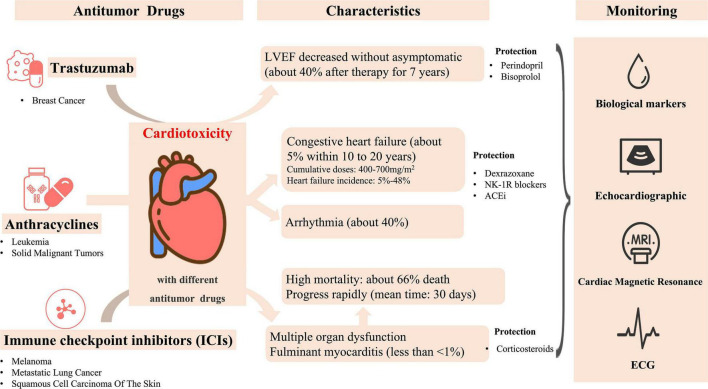
Some antitumor drugs and their characteristics of cardiotoxicity.

In animal experiments, Yi et al. revealed that trastuzumab combined irradiation caused more cardiotoxicity than irradiation or trastuzumab alone, which suggested that the concurrent management of trastuzumab and radiotherapy should be carefully made in clinical practice, and more attention is needed on cardiac safety (34) (Figure 1).

## Monitoring

The diagnosis of cardiotoxicity in patients undergoing cancer treatment before having clinical manifestations has a very positive effect on the prognosis. Therefore, patients with cancer undergoing chemotherapy are strongly recommended to undergo regular or even lifelong cardiac function monitoring. Cardiotoxicity monitoring commonly includes serum biological markers, ECG, echocardiography, cardiac magnetic resonance (CMR) and other methods such as endomyocardial biopsy (EMB) and cytokine measurements. Each of these detection methods has its own advantages and disadvantages. The disadvantages and the corresponding suggestions for the common methods for cardiotoxicity monitoring are shown in the Table 1.

**TABLE 1:** Common methods for monitoring cardiotoxicity.

Methods	Advantage	Disadvantage	Suggestion
Serum biomarkers	Cardiac troponin (cTnT and cTnI) and NPs are specific and sensitive biomarkers of cardiomyocyte damage	Traditional detection kits are less sensitive	Combine with imaging
ECG	Simple, non-invasive and inexpensive Holter can record changes over 24/48 h	Less specificity	As routine inspection
Echocardiography	Monitor the overall parameter of LV: EF, SF, LV wall stress, LV mass, LV thickness-to-size ratio, diastolic function, and GLS etc. Quick, easy to operate and good compliance	Without a description of the overall structure and subclinical myocardial changes cannot be detected	As routine inspection
CMR	Provide comprehensive information about structural, functional, tissue characteristics and myocardial perfusion Multiparameter and multisequence High repeatability	The cost of CMR is highly expensive for population-based screening High requirements for compliance	Preferred as far as possible

*cTnT, cardiac troponin T; cTnI, cardiac troponin I; NT, natriuretic peptide; ECG, electrocardiograph; CMR, cardiac magnetic resonance; LV, left ventricular; EF, ejection fraction; SF, fractional shortening; GLS, global longitudinal strain.*

### Biological Markers

Serum biomarkers are important for the baseline risk assessment and diagnosis of cardiovascular disease in cancer patients treated with potentially cardiotoxic drugs. The increase of cardiac biomarkers, especially cardiac troponin (cTn) and natriuretic peptide (NPs), can be used to guide the initiate of cardioprotective therapy during cancer treatment and monitor the responses of these protective therapy (35).

Antitumor therapy is often accompanied with troponin increasing, and patients with elevated troponin levels have a higher risk of left ventricular dysfunction (36). In a study of 204 patients treated with high-dose anthracyclines, 65 patients showed an increase in cTnI (>400 ng/L) and a continuous decrease in LVEF as measured consecutively before and after each treatment cycle (37). cTnI increasing were associated with progressive decline in LVEF in breast cancer patients (38). In addition, Auner et al. reported there were 15% of patients had increased cTnT (≥0.03 ng/mL) in patients with hematological malignancies, and the peak levels was observed on day 21 and was associated with a decrease in LVEF (39). For trastuzumab and HER2-targeted therapies, HER2-positive breast cancer (EBC) patients can detect an increase in hypersensitive troponin at 3 months after initiation of cancer therapy and could predict the development of left ventricular heart disease (40).

In addition, the elevation of BNP and NTroBNP during anthracycline treatment is also associated with the reduction of LVEF and poor prognosis (41). A cohort of 333 anthracycline-treated patients with different types of tumors showed that BNP > 100 pg/mL was a predictor of long-term heart failure, but not a risk factor for all-cause death. When BNP cutoff value was 30 ng/L, the negative predictive value of future development of heart failure was 98% (42). Among breast cancer patients treated with anthracyclines, De Iuliis et al. showed a significant increase in NTroBNP which associated with 1-year mortality (41). The continued increase of NTroBNP in the early stage after high-dose chemotherapy is also closely related to the development of cardiac dysfunction (43). Analysis of 555 cancer patients at diagnosis and before anticancer treatment also showed that Nt-proBNP and hs-cTnT were independent predictors of all-cause mortality (44).

A consensus from the Society for Immunotherapy of Cancer (SITC) Toxicity Management Working Group suggested that cardiac troponins screening should be performed in the first 12 weeks of ICI treatment (45). The assessment of creatine phosphokinase (CPK) may be useful because myocarditis is an inflammatory disease and may be associated with myositis (46), but this less sensitive marker may not rise significantly (47). Some novel serological markers such as myeloperoxidase, high-sensitivity C-reactive protein, SFLT-1, placental growth factor, Growth differentiation factor-15, galactose lectin-3, arginine nitric oxide metabolites, cardiac fatty acid binding proteins, glycogen phosphorylase BB and topoisomerase 2β are all increased to varying degrees after treatment with potentially cardiotoxic drugs (48, 49). Multi-marker strategies (combinations of multiple markers) may also further improve the ability to detect subclinical cardiotoxicity (35).

### Electrocardiogram

Electrocardiogram, as a routine evaluation method, can reflect the electrophysiological activity of the heart in time before, during and after tumor treatment. Tumor drugs may induce arrhythmias through a variety of ways (50). Although there is no specificity, different kinds of drugs with cardiovascular toxicity may have different manifestations on electrocardiogram, including sinus tachycardia, QT prolongation, ST-T segment changes, conduction block, etc. (51), among which QT prolongation is considered to be an important manifestation in the evaluation of cardiovascular duct toxicity. Andreu et al. conducted a systematic review of the incidence, diagnosis, and clinical outcomes of QT prolongation associated with tumor drug therapy and found that the weighted adjusted incidence of QT prolongation ranged from 0 to 22% in patients receiving conventional therapy (e.g., anthracyclines) (52).

Premature ventricular beats are the most common type of arrhythmia in patients treated with anthracyclines, with ventricular tachycardia occurring in approximately 73.9% of patients (53). Kilickap’s team conducted dynamic ECG monitoring for patients after 48 h of doxorubicin infusion, and the results showed that the rate of paroxysmal atrial fibrillation was 10.3% (54). The incidence of this arrhythmia was 6% when ECG monitoring was performed at each follow-up during the continuation of chemotherapy (55). In addition, arrhythmias may also occur in children treated with chemotherapy for cancer. Lipshultz et al.’s study found that 5% of children treated with doxorubicin developed unsustained ventricular tachycardia (56). In a study by Mulrooney et al., of 2,715 children survived from tumor disease, 290 (about 10%) had major ECG changes and 565 (23.3%) had minor changes, including atrial or ventricular premature beats, non-specific T wave or ST segment changes, low QRS and ECG axis deviation (57). Therefore, ECG monitoring is necessary for both adults and children before and after tumor treatment, especially for patients with high risk factors because timely detection of arrhythmias may improve the prognosis of patients.

### Echocardiography

Clinically, the most common parameters include EF, SF, LV wall stress, LV mass, rate of shortening of heart rhythm correction, LV thickness-to-size ratio, and diastolic function in the echocardiographic examination (7). In recent years, series of studies have confirmed myocardial strain abnormal earlier than cardiac function dysfunction in many cardiovascular diseases (58). Especially global longitudinal strain (GLS) provides prognostic information beyond EF among a broad range of cardiovascular diseases, from postmyocardial infarction (59) to aortic stenosis (60), as well as HF (61) and myocarditis (62, 63). For example, among patients with HF, each 1% improvement in GLS is associated with a 5% decreased risk of mortality (24, 61). The assessment of myocardial strain may also help to early detect cardiotoxicity in cancer patients (58), Ye et al. demonstrated that myocardial strain based on speckle-tracking echocardiography can predict further cardiotoxicity in patients receiving chemotherapy (64). A prospective study on 627 patients and implied the GLS was an optimal parameter of deformation for the early detection of subclinical LV dysfunction (64). A recent Strain Surveillance of Chemotherapy for Improving Cardiovascular Outcomes (SUCCOUR) randomized controlled trial (ANZ Clinical Trials ACTRN12614000341628) showed the patients in the LVEF-guided group who received cardiac protection had a greater decrease in LVEF at follow-up than those in the GLS-guided group, which further confirming that cardiac protection therapy guided by GLS as the main indicator can effectively delay cardiac function injury (65).

### Cardiac Magnetic Resonance

Although Echo is considered a routine test for cancer patients to monitor the cardiotoxicity, recent studies have shown that the LVEF measured by CMR has the highest repeatability compared to the LVEF measured by two-dimensional echocardiography (or myocardial deformation measured by both methods) in patients with cardiomyotoxicity due to cancer therapy (66, 67). The development of CMR fast scanning protocol and post-processing technology, and the automatic analysis of CMR images by machine learning algorithm not only improves the accuracy between observers, but also greatly reduces the image analysis time (42). Meanwhile, some studies revealed that precise evaluation by CMR may reduce the frequency of monitoring and increase the benefit of patients (43). All these studies indicated that CMR has a greater prospect in the monitor of cancer patients treatment. Besides, CMR plays an increasingly prominent role in the diagnosis of cardiotoxicity in multimodal imaging methods and it can better understand the underlying mechanisms of cardiovascular damage caused by cancer treatment, as well as other aspects related to cardiovascular structure and function (7).

#### Strain

Myocardial strain could be an alternative more sensitive imaging biomarkers than LVEF to earlier diagnosis and treatment of cardiotoxicity. The reduction in global longitudinal and circumferential strain assessed by CMR tissue-feature tracing is associated with subclinical decline in LVEF in cancer patients (68). The global circumferential strain (GCS) decreased while LVESV did not decrease significantly during chemotherapy, and the GCS was associated with LVEF measured 2 years later (69). The study by Jolly showed that circumferential strain assessed by CMR was associated with subclinical LVEF reduction in cancer patients undergoing cardiotoxic chemotherapy (70). Therefore, CMR assessment of myocardial strain may provide a more comprehensive and detailed parameter for patients with chemotherapy-induced cardiotoxicity.

#### T1/T2 Mapping

T1 mapping and T2 mapping technology on CMR is a promising non-invasive tool that can quantify myocardial tissue changes via changes in longitudinal and transverse relaxation, allowing for the early detection of cardiotoxicity (10). T2 mapping detects the production of edema in the myocardium, which is the earliest sign of anthracycline-induced cardiotoxicity. These changes occur in the reversible stage of myocardial disease, indicating that CMR markers can be used in tailor-made anthracycline therapy to monitoring cardiotoxicity (71). Elevated T1 mapping values were common in patients with ICI myocarditis. Thavendiranathan et al. reported that patients with higher T1 mapping values had signs of greater myocardial injury and T1 mapping values (for every 1-unit increase in z-score, hazard ratio: 1.44) were independently associated with subsequent MACE (72).

Quantitative T1 imaging can be used to calculate the myocardial extracellular volume fraction (ECV), a measure of microscopic myocardial remodeling that has been associated with underlying diffuse fibrosis (73). In recent study, CMR imaging were performed before and up to three times serially after cumulative anthracycline treatment in 27 women with breast cancer. Those patients undergoing anthracycline therapy had significant reductions in LVEF and LV mass, and mean ECV had increased by 0.037 to 0.36 ± 0.04 (p = 0.004) (74).

#### Late Gadolinium Enhancement

Myocardial fibrosis can be detected non-invasively by late gadolinium enhancement (LGE) with CMR, which is the priority imaging test for the diagnosis and risk prediction in myocarditis of other etiologies (75–81). But Zhang et al. analyzed the CMR results of 103 patients diagnosed with ICI-associated myocarditis, and they found that LGE present in more than 80% of patients with non-ICI-related myocarditis but occurs in less than 50% of patients with ICI-associated myocarditis, therefore, clinicians should use more CMR indicators to diagnose or exclude ICI-associated myocarditis rather than use global LGE only (82). When two or more suspicious indicators, such as inducible perfusion deficit, regional or global dysfunction, edema, necrosis, scar, and pericarditis are monitored by CMR, ICI-related cardiotoxicity may be indicated (83). In a recent study, although the global LGE was less frequent in patients with ICI-myocarditis than those with viral myocarditis, septal and midwall layer LGE was more common. Septal LGE was the only CMR predictor of MACE at 1 year after adjustment for peak troponin (84).

**FIGURE 2:**
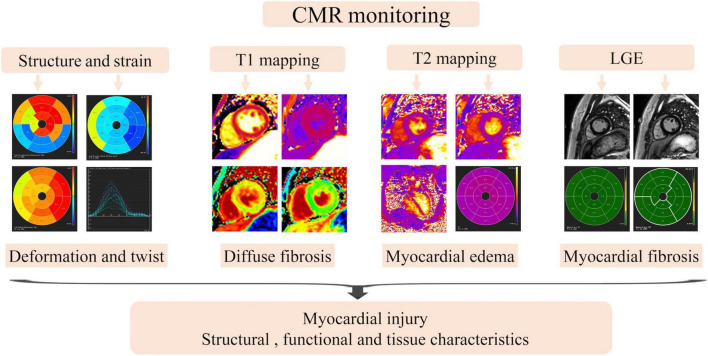
The monitoring of myocardial injury by multi-parameter of cardiac magnetic resonance.

Cardiac magnetic resonance are helpful in individuals whose echocardiographic techniques are not feasible or whose results are suboptimal, and provide more information about myocardial perfusion. However, the cost of CMR is extremely expensive for a population-based screening, and comprehensive multi-parametric CMR tissue studies are currently unavailable (71). In recent years, research in the field of CMR is developing rapidly, and CMR might become the best means to monitor cardiotoxicity in the future due to its multi-sequence characteristics (Figure 2).

### Other Methods

Cytokines, inflammatory factors, and endocardial biopsies can also be used to monitor myocardial toxicity. The cytokine’s sensitivity to inflammation makes it useful for monitoring cardiotoxicity. An animal experiment showed that the cardiac tissue levels of TNF-α, IL-6, and IL-1β in the DOX-treated group were significantly increased as compared to the normal group rats (85). Ahmed et al. also found that administration of trastuzumab resulted in significant increase in cardiac tissue IL-6 and TGF-b1 expression compared to the control group (1362.5 ± 18.5 vs. 211.2 ± 6.4 pg/g tissue; 11.32 ± 0.3 vs. 3.42 ± 0.12 pg/μg protein, respectively) in mice (86).

Myocarditis is one of the important manifestations of ICI related cardiotoxicity. Recently Vincenzo et al. found that the expression of IL-6 and IL-1 in breast cancer cells and cardiomyocytes exposed to ipilimumab significantly increased when in high glucose state, suggesting the protective role of low glucose in immune-suppression and cardiotoxicity (87).

Endomyocardial biopsy is available to detect tissue damage, primarily for rejection monitoring after cardiac transplantation and also have an important complementary role to the clinical assessment in establishing the diagnosis of diverse cardiac disorders (88), but the use is declining due to its invasive and costly (89, 90). EMB may be considered if CMR or 18 F-fluorodeoxyglucose PET-computed tomography yield uncertain findings and/or the patients cannot undergo non-invasive assessment due to haemodynamically instability (82).

## Prevention of Cardiotoxicity

### Prevention of Anthracycline

Dexrazoxane (DEX) is an iron chelating agent that minimizes cardiotoxicity by limiting the production and accumulation of ROS in myocardium and preventing the interaction between antitumor drugs and topoisomerase II (91). Dex is mainly used in advanced and/or metastatic adult breast cancer patients with cumulative doxorubicin doses up to 300 mg/m^2^ or epirubicin cumulative doses up to 540 mg/m^2^. Ganatra et al. observed clinical outcomes in five patients with preexisting asymptomatic left ventricular systolic dysfunction who required chemotherapy with anthracycline, in combination with off-label treatment with Dexrazoxane 30 min before each anthracycline dose. All five patients treated with Dex successfully completed chemotherapy as planned, with mean LVEF decreasing from 39% at baseline to 34% after chemotherapy, without symptomatic heart failure or elevated biomarkers (cardiac troponin I or brain natriuretic peptide). In three patients who were not treated with Dexrazoxane, the LVEF decreased from 42.5% at baseline to 18% after treatment. All patients developed symptomatic heart failure requiring hospitalization and diuretic therapy, and two died of cardiogenic shock and multiple organ failure. Therefore, this study suggested that Dex can reduce the cardiotoxicity induced by anthracyclines in adults with existing cardiomyopathy and tumors (92). Lisa et al. showed that dextroimide prevents left ventricular dysfunction and heart failure in patients with osteosarcoma treated with high doses of anthracyclines, especially in girls, by post-evaluation in patients with osteosarcoma treated with dextroimide (93). Dewilde et al. thought that no matter which healthcare system they receive treatment in, Dex protection is a cost-effective way to prevent cardiotoxicity of anthracyclines in children with sarcomas or hematological malignancies and these benefits persisted when patients received cumulative doses of anthracyclines greater than 250 mg/m^2^ (94).

Otherwise, various of anti-cardiotoxicity drugs has been widely investigated. For instance, neurokinin-1 receptor blockers can reduce adriamycin-induced cardiac fibrosis and to prevent possible LV damage (95). The ACEi inhibitor administration improved the impaired heart function in the patient with cardiotoxicity induced by doxorubicin or mitoxantrone (4, 96).

### Prevention of Immune Checkpoint Inhibitors

For the cardiac protection treatment, corticosteroids are currently the main first-line treatment for ICI-induced myocarditis. However, considering the lack of evidence, the suggestion of initial glucocorticoid doses and treatment strategies are varies greatly. A recent retrospective observational study emphasized that an increased initial dose (intravenous methylprednisolone, 1,000 mg/day) and the early use of corticosteroids are associated with improved cardiac prognosis in ICI-related myocarditis. This previous study supported that the initial dose of corticosteroids is inversely related to the occurrence of major adverse cardiovascular events (MACE), the rate of MACE are 61.9, 54.6, and 22.0% respectively in low, medium and high dose initially (97). Patients receiving corticosteroids within 24 h after admission (7.0%) had a lower MACE occurrence rate than those who received it between 24 and 72 h (34.3%) and >72 h (85.1%). However, the effect of high-dose corticosteroids on the outcome of patients with cancer who take ICI remains controversial. In addition, some studies suggest that if the symptoms of myocarditis do not immediately respond to steroids, upgrading to other immunosuppressive drugs, such as infliximab, mycophenolate mofetil, and anti-thymocyte globulin, may be necessary (98).

### Prevention of Trastuzumab

The specific mechanism of trastuzumab related cardiotoxicity remains unclear (99, 100). There were study reported the angiotensin-converting enzyme inhibitors (ACEi) and beta-blockers might prevent trastuzumab cardiotoxicity (99, 101, 102). However, the clear curative effects of these drugs still uncertain. Edith et al. (103). used perindopril and bisoprolol to protect the myocardium and found that these drugs were well tolerated by patients with HER2-positive early breast cancer; both drugs mitigated the LVEF decline associated with trastuzumab but did not prevent left ventricular remodeling, thus the long-term significance of LV remodeling in that study is unclear, and the cardiovascular risk factors in patient cohort are fewer than clinical practice, which require further investigation to confirm these drugs’ effects in chemotherapeutic-related cardiotoxicity.

## Conclusion

Oncological cardiology is an emerging discipline involving oncology, cardiovascular, imaging, laboratory, and other fields. With the continuous in-depth research on antitumor drugs, various chemotherapeutic and new targeted drugs are being used widely in clinical, and their adverse effects cannot be ignored. Such adverse events may be the major causes of death. Cardiotoxicity based on chemotherapy drugs and ICIs is mainly manifested as various types of cardiac dysfunction, HF and myocarditis and may be associated with high morbidity and mortality. Therefore, early detection of cardiotoxicity facilitated by these monitor methods especially the advanced multimodality imaging techniques summarized in this review will permit intervention at an earlier stage, which is crucial for the improvement of patient’s quality of life and decrease in mortality risk. The future development of oncological cardiology may require more attention to high-risk populations, cardiac function monitoring, and preventive and therapeutic administration. Furthermore, the mechanism of cardiotoxicity caused by various traditional chemotherapy agents and newer targeted cancer therapies should be researched further to determine ways on how to protect patients with cancer from cardiotoxicity.

## Author Contributions

WH and RX wrote the manuscript. BZ and CL provided a detailed guidance throughout the manuscript. HX, XG, and YG responsibility for the integrity of the work as a whole from inception to published manuscript. All authors contributed to the article and approved the submitted version.

## Conflict of Interest

The authors declare that the research was conducted in the absence of any commercial or financial relationships that could be construed as a potential conflict of interest.

## Publisher’s Note

All claims expressed in this article are solely those of the authors and do not necessarily represent those of their affiliated organizations, or those of the publisher, the editors and the reviewers. Any product that may be evaluated in this article, or claim that may be made by its manufacturer, is not guaranteed or endorsed by the publisher.
